# Production of Bacterial Cellulose Aerogels With Improved Physico-Mechanical Properties and Antibacterial Effect

**DOI:** 10.3389/fbioe.2020.603407

**Published:** 2020-12-02

**Authors:** Viktor V. Revin, Natalia B. Nazarova, Ekaterina E. Tsareva, Elena V. Liyaskina, Vadim D. Revin, Nikolay A. Pestov

**Affiliations:** Department of Biotechnology, Bioengineering and Biochemistry, National Research Ogarev Mordovia State University, Saransk, Russia

**Keywords:** biocomposites, antibacterial activity, bacterial cellulose, TEMPO oxidation, aerogels

## Abstract

Aerogels have gained significant interest in recent decades because of their unique properties such as high porosity, low density, high surface area, and excellent heat and noise insulation. However, their high cost and low mechanical strength limit their practical application. We developed appropriate conditions to produce aerogels with controlled density, high mechanical strength, and thermal characteristics from bacterial cellulose (BC) synthesized by the strain *Komagataeibacter sucrofermentans* H-110. Aerogels produced using TEMPO oxidized BC (OBC) exhibited high mechanical strength and lower shrinkage than those from native bacterial cellulose (NBC). Compared to the NBC, the use of TEMPO-oxidized BC with oxidation degrees (OD) of 1.44 and 3.04% led to the reduction of shrinkage of the aerogels from 41.02 to 17.08%. The strength of the aerogel produced from the TEMPO-oxidized BC with an oxidation degree of 1.44% was twice that of the aerogel produced from NBC. The addition of Mg^2+^ at concentrations of 20 and 40 mM during the preparation of the aerogels increased the strength of the aerogels by 4.9 times. The combined use of TEMPO-oxidized BC and Mg^2+^ allowed pore size reduction from 1,375 to 197.4 μm on the outer part of the aerogels, thereby decreasing the thermal conductivity coefficient from 0.036 to 0.0176 W/(m•K). Furthermore, novel biocomposites prepared from the aerogels based on NBC and OBC and sodium fusidate, which have high antibiotic activity against *Staphylococcus aureus*, were obtained. Owing to their antibacterial properties, these aerogels can be used as functional biomaterials in a wide range of applications such as in tissue engineering and fabrication of wound dressing materials.

## Introduction

Aerogels are one of the most interesting materials of the twenty-first century (Barrios et al., 2019). They have gained considerable interest because of their unique properties such as high porosity, low density, high surface area, and excellent heat and noise insulations. However, high production costs and low mechanical strength limit their practical application (Nita et al., 2020).

Aerogels are highly porous nanostructured materials that were first created by Kistler in 1931 (Kistler, 1931; Nita et al., 2020). They can be produced by two methods–supercritical drying or freeze-drying. In both, the microporous structure of the material is preserved during drying. Therefore, this material has a high porosity, an extremely low density, and a high specific surface area. The first aerogel produced by Kistler was a silica aerogel obtained through supercritical drying. The precursors used to produce silica aerogels are toxic, and the resulting aerogel is not biodegradable and has unsatisfactory strength (Katti et al., 2006). The high production cost for such an aerogel combined with the above disadvantages, is the main reason that this material is not widely used.

Aerogels can be inorganic, organic, or hybrid (Novak et al., 1994; Nita et al., 2020) depending on the material used for their production. Organic aerogels are obtained from biopolymers that are based on renewable raw materials. The most common biopolymer on Earth is cellulose. In applications that require biodegradability and biocompatibility, cellulose aerogels can be an excellent alternative to silica aerogels. These applications include medicinal purposes—where the aerogel can be used for directed drug delivery—and tissue engineering and regenerative medicine (Bhandari et al., 2017; Nita et al., 2020; Zheng et al., 2020). The high sorption capacity of cellulose aerogels for heavy metals and toxic organic compounds allows them to be used for removing undesirable pollutants from the environment (Nguyen et al., 2014; Maleki, 2016; Wang et al., 2019). Owing to the formation of an open-cell structure the aerogel can act as an effective heat-insulator (Nguyen et al., 2014).

Cellulose of various origins, such as the common vegetable cellulose from wood (Gupta et al., 2018), plant cellulose, and bacterial cellulose (BC) (Pircher et al., 2016; Revin et al., 2019), can be used to produce aerogels (Long et al., 2018). BC is an extracellular product of the metabolism of certain bacteria, and the main function of this polymer is to deliver bacterial cells to the upper layers of the nutrient medium, wherein their oxygen demand is met. The most promising producers of BC are bacteria of the genus *Komagataeibacter* (formerly the genus *Gluconacetobacter*) (Yamada et al., 2011). Moreover, BC has the same chemical composition as plant cellulose; however, its structure differs from the plant polymer. These differences arise because BC is synthesized in a chemically pure form, the thickness of its fibrils is much less (20–80 nm) than that of the plant polymer, and has a higher degree of crystallinity (Revin et al., 2018). BC has several biorelevant properties including biological compatibility, high mechanical strength, and high sorption capacity. This makes it a unique carrier matrix for enzymes, cells, and various medications, particularly with hemostatic, antibacterial, and regenerative effects.

Recently, BC has attracted significant attention because it can be used to produce a wide range of functional and structural materials (Choi and Shin, 2020). It has excellent potential for applications in medicine such as a biomaterial for tissue engineering (Carvalho et al., 2019; Hickey and Pelling, 2019; Luo et al., 2019), wound dressing (Sulaeva et al., 2015; Carvalho et al., 2019; Portela et al., 2019; Teixeira et al., 2020), and controlled drug delivery (Carvalho et al., 2019). Furthermore, BC can be used in dietetics as a carrier of additives for balanced nutrition, in industrial electronics to produce optically transparent compounds with an ultra-low thermal expansion coefficient, and in manufacturing of acoustic diaphragms. It is a promising source for obtaining nanocrystalline cellulose, and biocomposite materials (Sharma et al., 2019; Choi and Shin, 2020). Currently, it is prevalent in the production of several biocomposites that have antibacterial and hemostatic properties. Biocomposites with high antibacterial activity are produced based on BC and silver nanoparticles (Wu et al., 2018), copper (Ma et al., 2016), zinc (Jebel and Almasi, 2016), and a few antibiotics such as tetracycline, gentamicin, and amoxicillin (Kaplan et al., 2014; Shao et al., 2016). Biocompatible Au nanoclusters (AuNCs) were synthesized and added to a cellulose nanofibril dispersion to prepare a novel antibacterial film (Wang et al., 2020). A previous study reported the synthesis of biocomposites that are based on BC and chitosan (Zang et al., 2016). A review by Zheng et al. covers the challenges in obtaining medical biomaterials in the form of aerogels for wound coverings, bone regeneration, and directed drug delivery (Zheng et al., 2020).

Most of the practical applications of aerogels require certain strength characteristics. Hence, it is particularly important to identify and understand the factors that affect the strength of the aerogels. The strength characteristics of a BC aerogel depend on both its density and microporous structure (Revin et al., 2019). Chemical modification methods are widely used to adjust the properties of cellulose and its derivative materials (Dufresne, 2013). Cellulose oxidation is a common method for modifying the properties of this biopolymer. There are two main methods to oxidize BC: TEMPO (2,2,6,6-tetramethylpiperidin-1-yl)oxyl) oxidation, in which the primary hydroxymethyl group is selectively oxidized to a carboxyl group, and periodate oxidation, in which the C2–C3 bond is broken to form two aldehyde groups.

The purpose of this work is to obtain BC aerogels with specified properties, such as controllable density, strength, and corresponding typical characteristics. Considering the properties of aerogels, another important task is to obtain biocomposites in aerogel forms for biomedical applications, which is one of the novel contributions of this work.

## Materials and Methods

### Preparation of BC

BC was produced in a static culture medium by *Komagataeibacter sucrofermentans* H-110, which was isolated from Kombucha tea and identified by sequencing the amplified product of 16S rRNA (Revin et al., 2020). A strain was deposited in the Russian National Collection of Industrial Microorganisms (VKPM) (Accession No. VKPM: B-11267). For the production of BC, a Hestrin and Schramm (HS) medium that contained glucose (20 g/L), peptone (5 g/L), yeast extract (5 g/L), citric acid (1.15 g/L), and disodium hydrogen phosphate (2.7 g/L) at a pH of 6.0 was used. The culture medium was autoclaved for 20 min at 120°C. Further, the medium was inoculated with 10% (v/v) inoculum. To prepare the inoculum, *K. sucrofermentans*H-110 was transferred aseptically from an agar plate to a 250-ml Erlenmeyer flask containing 100 ml of culture medium and incubated in a shaker incubator (Model ES-20/60, BIOSAN, Latvia) at 28°C for 24 h at 250 rpm. The BC was produced in static conditions at 28°C for 5 days. After the incubation, the BC was collected, washed thoroughly with de-ionized water to remove medium components, and treated with 1% (w/v) sodium hydroxide solution at 80°C for 1 h to eliminate the bacterial cells. Further, the BC was rinsed extensively with 6% (v/v) acetic acid and then de-ionized water until the pH became neutral. If necessary, the purified BC was dried to a constant weight at 60°C.

### TEMPO Oxidation of Bacterial Cellulose

In this study, 10 ml of distilled water containing 48 mg NaBr and 0.2 mg TEMPO was added to 8 g of the BC gel film. The pH of the solution was adjusted to 10.0 by adding 0.5 M NaOH. Further, 3 ml of 5% NaClO was added to the solution. The reaction was performed at 22°C for 15, 30, 45 and 60 min. The reaction was stopped by adding 5 ml ethanol to decompose the residual NaOCl. After oxidation, to remove the reagents, the gel film of oxidized BC was thoroughly washed with distilled water until a pH value of 5.5 was reached. This pH value should remain stable for 3 h.

The oxidation degree (OD) of BC was determined by conductometric titrations using a S70-K SevenMulti conductivity meter. Eight gram of BC gel film was suspended into 50 mL of 0.01 M hydrochloric acid solution. After 60 min of stirring, the suspensions were titrated with 0.01 M NaOH. The relative amount of carboxyl groups on the BC, which is indicated by OD, was calculated using the following equation (Perez et al., 2003):

$$\begin{array}{l} OD=162\left(V2-V1\right)c{\left[w-36\left(V2-V1\right)c\right]}^{-1} \end{array}$$

where V2 is the amount of NaOH (in L) required for the titration of excess of HCl and weak acid corresponding to the carboxyl content and V1 is the amount of NaOH (in L) required for the titration of excess of HCl, c is the NaOH concentration (mol/L), and w is the weight of oven-dried sample (g).

### Aerogel Production

The BC aerogels were obtained using the freeze-drying method. The hydrogel was produced by grinding the cellulose obtained from the cultivated bacteria under static conditions using a laboratory homogenizer for 5 min. The BC hydrogel was frozen in a single step in a foil with the required shape. The resulting BC hydrogel was placed in a −50°C freezer for 24 h. Freeze-drying was conducted using a FreeZone Plus freeze-dryer lyophilizer (Labconco, USA) for 72 h.

To obtain aerogels from the TEMPO-oxidized BC containing divalent metals, an appropriate amount of salts of the corresponding metals was added to the obtained BC hydrogel until the required concentration was obtained. The following salts were used as the sources of the divalent metals: MgSO_4_, MnSO_4_, CoSO_4_, ZnSO_4_, CuSO_4_, NiSO_4_, CaCl_2_, and ZnSO_4_.

### Production of Biocomposites Based on BC Aerogels and Sodium Fusidate (SF)

Biocomposites based on the BC aerogels and SF were obtained using the freeze-drying of hydrogels from native bacterial cellulose (NBC) and oxidized bacterial cellulose (OBC) with an OD of 1.44%. To impart the antimicrobial properties, the antibiotic SF, at concentrations of 500 μg/g, was added to the hydrogel. To obtain the aerogels, the hydrogels were frozen in a low-temperature cooler MDF-U53V (SANYO, Japan) at −50 °C for 24 h, and further dried on a FreeZone Plus freeze-dryer lyophilizer (Labconco, USA). The obtained aerogels were cut into round pieces with a diameter of 10 mm, weighing 0.002 g and containing 100 μg of SF. The final composites were identified as NBC/SF_100_, and OBC/SF_100_.

### Determination of Aerogel Properties

The density of the aerogels was determined by volume and weight measurement. The aerogel volume was determined based on its linear dimensions.

The strength of aerogels from NBC and OBC was determined using digital force gauge ISF-DF5, “Insize.” For this study, cylindrical aerogels with a diameter of 14 mm and a height of 10 mm were used.

Scanning electron microscopy (SEM) of the BC aerogels was performed using a Quanta 200 I 3D FEI scanning electron microscope (USA). Average pore sizes were determined using the image analysis software FEI xT microscope server. At least 100 pores on each SEM image were measured. To determine the pore area, the total area occupied by the pores having the same size was calculated. The area of a single pore was determined based on the linear dimensions of each pore.

The shrinkage of the aerogels was determined using the following equation:

$$\begin{array}{l} Shrinkage=\frac{\left({\text{V}}_{initial}-V_{final}\right)}{V_{initial}}\times 100 \end{array}$$

where *V*_*initial*_ and *V*_*final*_ are the volumes of the BC hydrogel that was used to produce the aerogel and the aerogel obtained after freeze-drying, respectively.

The porosity of the aerogels was determined using the following equation:

$$\begin{array}{l} Porosity\left(\%\right)=\left(1-\frac{\rho_{apparent}}{\rho_{actual}}\right)\times 100 \end{array}$$

where ρ_*apparent*_ is the apparent density that was determined based on the linear size of the aerogel, and ρ_*actual*_ is the density of BC, which is 1.38 g/cm^3^ (Wang et al., 2017).

The thermal conductivity coefficient of the sample (λ) was determined using the following equation:

$$\begin{array}{l} \lambda =\frac{dq}{\Delta T} \end{array}$$

where *d* is the thickness of the sample (in m), *q* is the heat flow density, and ΔT is the temperature difference between the sample facets.

The measurement of the heat flow density and the temperatures of the opposite face sides was performed using a digital device for measuring heat flow density (HFM-2). We considered the heat flow, which flowed through a test sample with dimensions of 60 × 40 × 10 mm, to be established if the values of the sample thermal conductivity, calculated from the results of five consecutive measurements, differed from each other by <1%.

Thermogravimetric analysis was performed using a TG 209 F1 Libra thermobalance (Netzsch, Germany).

### Fourier-Transform Infrared (FTIR) Spectra of BC

The BC was freeze-dried and crushed into a powder form, mixed with potassium bromide, and pressed into a small tablet that was subjected to FTIR spectroscopy using an FTIR spectrometer IRPrestige-21 (Shimadzu, Japan) in the absorption mode. For each sample, 32 scans at a resolution of 4 cm^−1^ at wavenumbers ranging from 4,000 to 400 cm^−1^ were collected.

### AFM Micrographs of BC

The surface morphologies of BC were studied by contact atomic force microscopy (AFM) using an SPM 9600 (Shimadzu, Japan) microscope. The BC samples were air-dried on a glass slide in the form of a thin film. A silicon nitride cantilever with a nominal radius of 2 nm of the pyramidal tip was used. The scan rates ranged between 0.6 and 1.0 Hz/s. An image resolution of 256 × 256 points was set.

### Antibacterial Activity

The antibacterial activities of the prepared aerogels were studied by the disc diffusion method against *Staphylococcus aureus* 209 P from VKPM (VKPM B-6646, ATCC 6538P). The antibacterial capacity is determined by measuring the diameter of the clear zone of inhibition around the samples after 18 h of incubation at 37°C.

### *In vitro* Release Assays

The release behavior of SF was studied in 100 mL of phosphate buffer solution at pH 7.4. The quantitative analysis of SF was performed using a microbiological method (Hikalx et al., 1982). An agar plate diffusion technique was employed using *S. aureus*209 P as the test organism. After the agar had solidified, wells (10-mm i.d.) were punched out, and 0.1 mL of a standard or test dilution was applied. Standard dilutions contained 100, 200, 300, 400, 500, 600, 700, 800, 900, and 1,000 μg/mL of SF. The zone of inhibition diameter was measured after 18 h of incubation at 37°C.

### Cytotoxicity Tests

The study was made on mouse fibroblast cell culture L929 (obtained from tissue culture collections of D.I. Ivanovsky Institute of Virology, Russia). The cells were cultured in Dulbecco's modified Eagle's medium (DMEM) (Paneko, Russia) with 10% fetal bovine serum (FBS) (HyClone, USA) and antibiotics (penicillin, streptomycin) under the standard conditions: 5% CO_2_ atmosphere, *t* = 37°C, 5% humidity in an incubator MCO-170M (Sanyo, Japan). For the study, cells in the exponential growth phase were dispersed in a 96-well plate (5 × 10^3^ cells/well). After 24 h, the medium in the 96-well plate was replaced by fresh medium containing the aerogels extracts followed by incubation for 24 h. The aerogels NBC/SF_100_ and OBC/SF_100_ were extracted by immersion of the aerogels in DMEM at 37°C for 24 h. Wells containing only the cells were used as control. After the incubation time the morphological structures was observed by an inverted optical microscope (Micromed, Russia). The cytotoxicity was measured using the MTT assay method. The medium was replaced with the fresh one with 5 mg/mL dimethyl thiazolyl diphenyl (MTT) solution. After 4 h incubation time the medium was removed, 150 μL DMSO was added. Optical density was measured on a microplate reader EFOS 9305 (Russia) at a wavelength of 492 nm with a reference wavelength of 620 nm. Cell viability was determined as the ratio of the optical density of the sample to the control expressed as a percentage.

### Statistical Analysis

All the presented data are averages of at least three rounds of experiments, which were performed with three to six replicates of the mean. The standard deviations of the means were calculated using Microsoft Excel 2013 (Microsoft Corporation, Redmond, USA). The obtained data were statistically analyzed by a student *t*-test: two-sample assuming equal variances. The differences were considered significant at the level of *p* < 0.05.

## Results

### Morphology of BC

**Figure 1:**
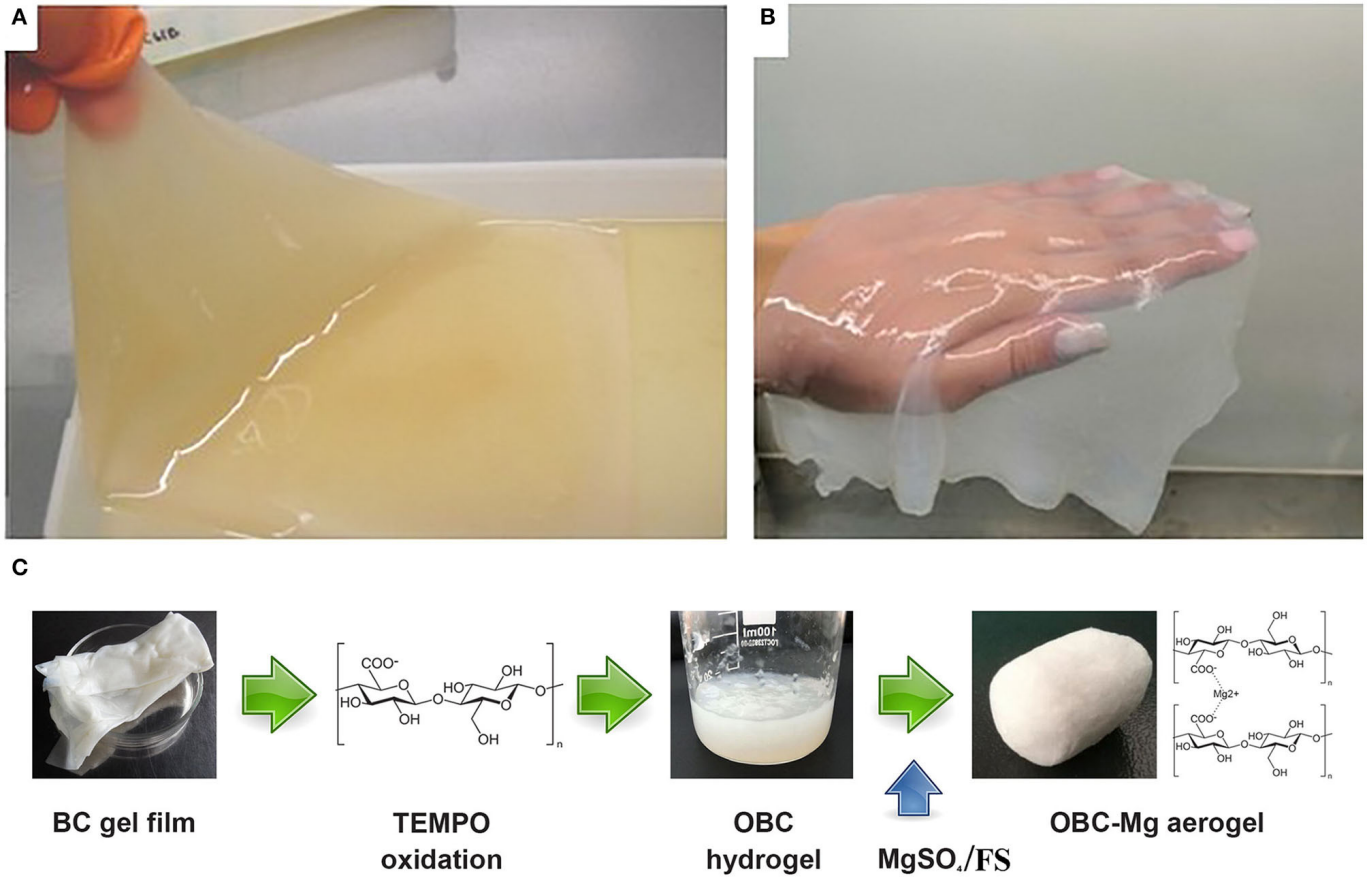
A BC gel film on the surface of the HS medium **(A)**, after its purification **(B)**, and scheme for producing NBC or OBC aerogels **(C)**.

The macrostructure morphology of BC varied depending on the different culture methods (Wang et al., 2019). In an agitated culture, the cellulose was synthesized in deep medium in the form of fibrous suspensions, pellets, and irregular masses (Revin et al., 2018). In a stationary culture, a gelatinous cellulose film was formed at the air/liquid interface of the medium. When cultivating bacterium *K. sucrofermentans* H-110 at static conditions in the HS growing medium for 5 days, a BC gel film was formed on the surface of the medium (Figure 1A). After treatment, the gel film became colorless and transparent (Figure 1B). Figure 1C shows a general scheme for producing NBC or OBC aerogels containing Mg^2+^ or SF.

**Figure 2:**
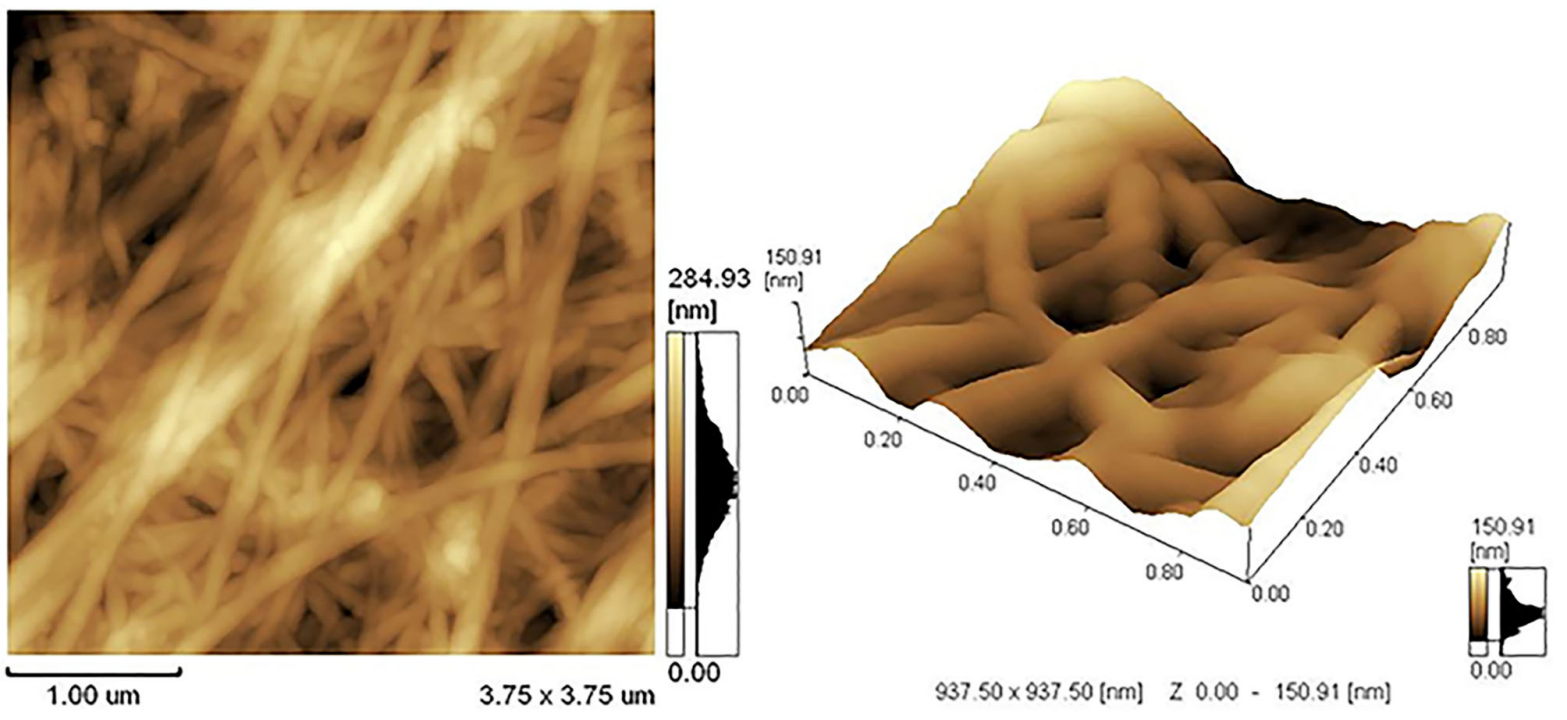
AFM image of the BC.

AFM was used to study the microscopic details of the biopolymer. The micromorphology of the BC, that exhibited a nanoporous three-dimensional (3D) network structure with a random arrangement of the ribbon-shaped fibrils, is shown in Figure 2. The average thickness of the BC fibrils formed on the standard HS medium was 50–90 nm.

### Oxidation of BC

**Figure 3:**
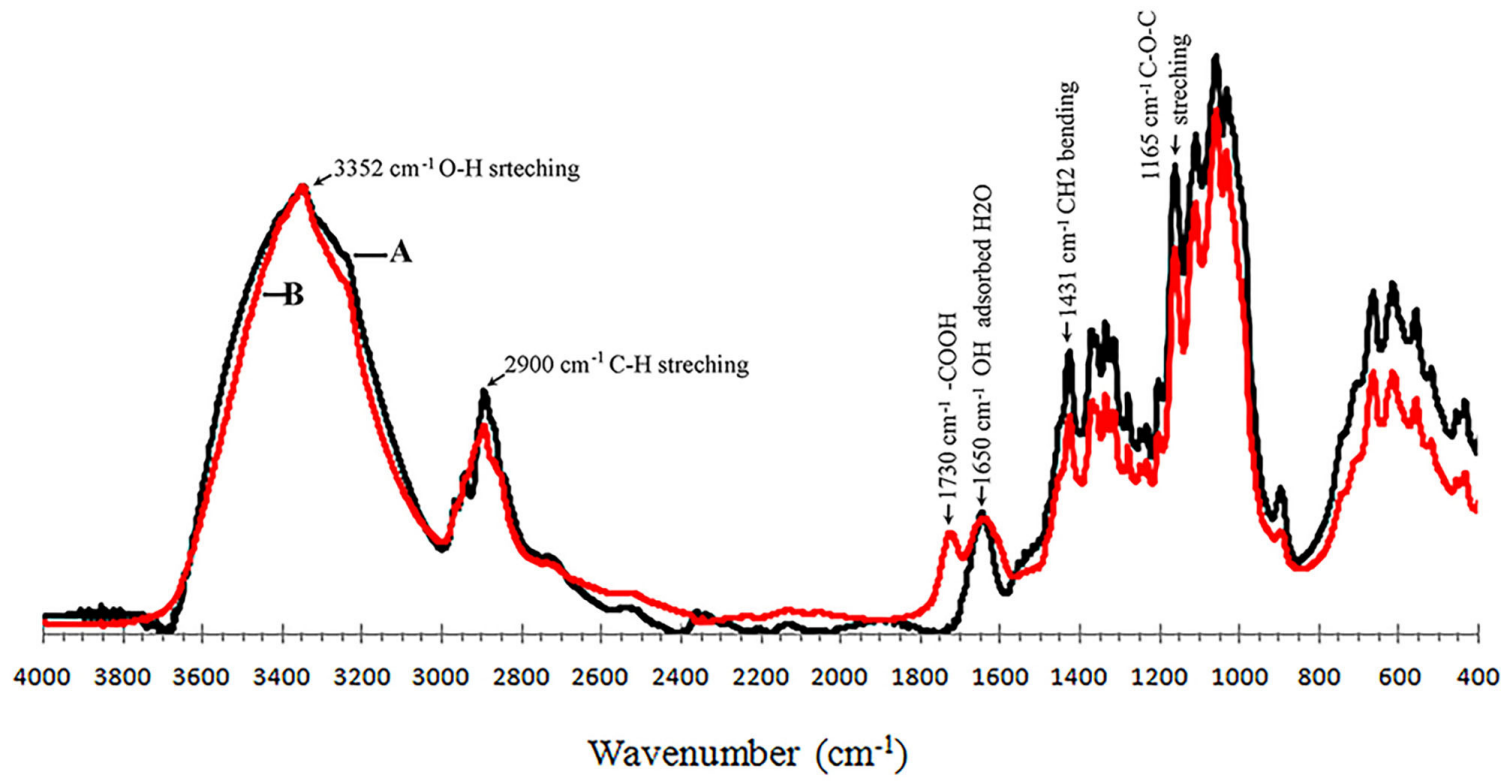
IR spectra of purified NBC **(A)** and TEMPO-oxidized BC **(B)**.

BC aerogels can be obtained from BC synthesized under static and dynamic cultivation conditions (Revin et al., 2019). An important parameter that determines the consumer qualities of the aerogels is the strength of the material. These characteristics strongly depend on the density of the aerogels obtained from the BC (Revin et al., 2019). Therefore, it is required that the strength of the BC aerogels be increased without increasing their densities. To modify the properties of the BC, chemical modification methods can be used. In this work, TEMPO oxidation was used for the BC gel film modification. The oxidation of cellulose using a TEMPO reagent leads to a specific oxidation of the primary –CH_2_OH group of the glucopyranose unit to –COOH. The qualitative analysis of the TEMPO oxidation of the BC was determined by infrared (IR) spectroscopy (Figure 3).

The IR spectrum of the native BC gel film showed absorption peaks that are typical of this polymer. The absorption peak in the 3,000–3,600 cm^−1^ region can be distinguished, and corresponds to the stretching vibrations of the cellulose hydroxyl groups involved in the formation of hydrogen bonds. A wide and less-intense band at 2,800–3,000 cm^−1^ corresponds to the stretching vibrations of the C–H bonds of the cellulose methylene groups (Kondo and Sawatari, 1996). The peak in the 1,650 cm^−1^ area is due to the deformation vibrations of the –OH groups from adsorbed water. The peak at 1,431 cm^−1^ is caused by the symmetric –CH_2_ bending (Cael et al., 1975). The intense band at 1,000–1,047 cm^−1^ corresponds to the valence vibrations of the C–O bonds. The peak in the 1,162–1,125 cm^−1^ region is caused by the asymmetric bending of C–O–C (Oh et al., 2005). The oxidation of BC leads to the appearance of the –COOH groups in this polymer. The absorption of the –COOH group depends on its protonation state. When the carboxyl group is protonated, a peak appears at 1,730 cm^−1^ (Naumann et al., 1982; Jiang et al., 2004).

The TEMPO oxidation of the BC led to the appearance of a peak that exhibits the IR spectrum characteristics of the –COOH group. Particularly, a pronounced peak at 1,730 cm^−1^, which indicates the successful oxidation of the BC, was observed after the transition of the –COOH groups in the protonated state.

### Influence of the OD of BC on the Properties of Produced Aerogels

By changing the oxidation conditions of the BC, it is possible to obtain samples of the oxidized BC gel film with different ODs. During the oxidation of the BC, samples of the OBC were obtained with ODs of 1.03, 1.44, 3.04, and 4.15%. The ODs were determined by conductometric titrations.

Obtaining aerogels through freeze-drying was accompanied by a decrease in the volume of the aerogels, which is called aerogel shrinkage (Long et al., 2018). In the freeze-drying method for producing aerogels from the NBC, the shrinkage of the resulting material reached 41% (Table 1).

**Table 1:** Effect of BC gel film oxidation on the aerogel properties.

**Parameters**	**Aerogel produced from NBC**	**Aerogel produced from TEMPO-oxidized BC with an oxidation degree, %**			
		**1.03**	**1.44**	**3.04**	**4.15**
Volume, cm^3^	2.654 ± 0.013	3.077 ± 0.015	3.697 ± 0.018	3.699 ± 0.018	3.543 ± 0.017
Porosity, %	99.16 ± 0.10	99.25 ± 0.09	99.42 ± 0.09	99.39 ± 0.09	99.32 ± 0.09
Shrinkage, %	41.02	31.62	17.84	17.80	21.27
	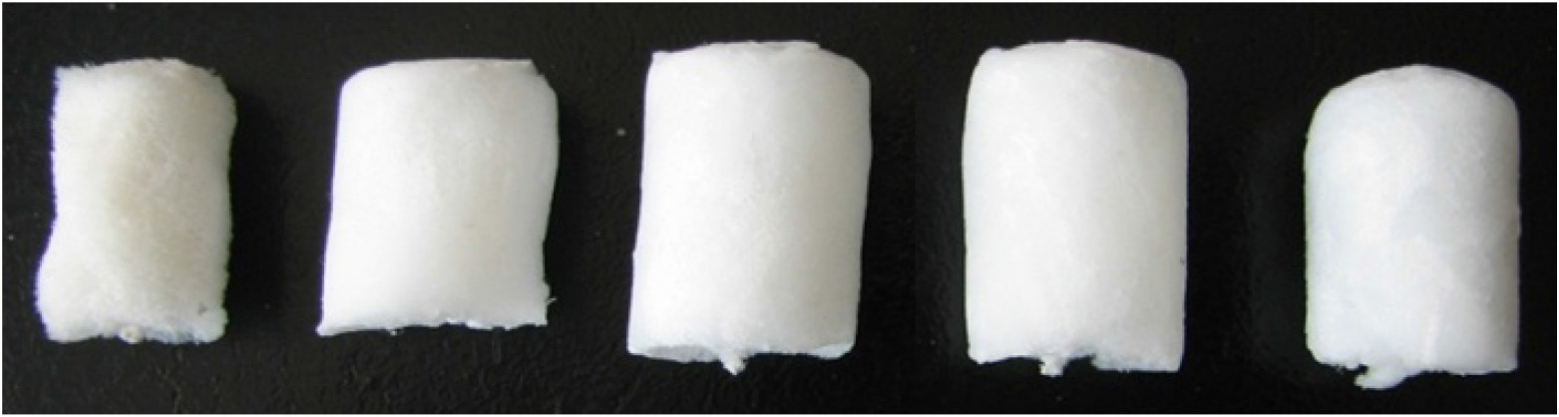				
	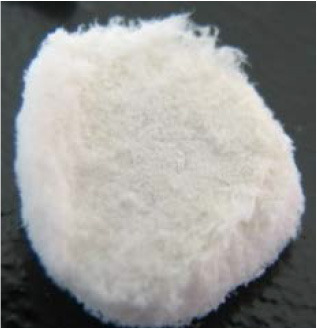	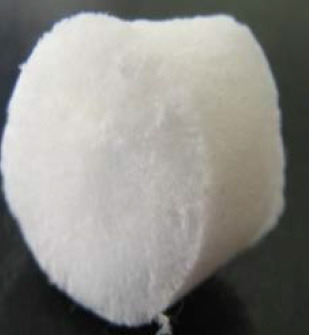	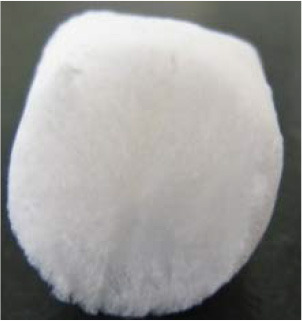	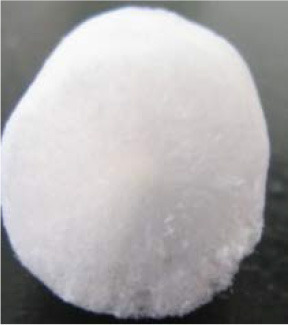	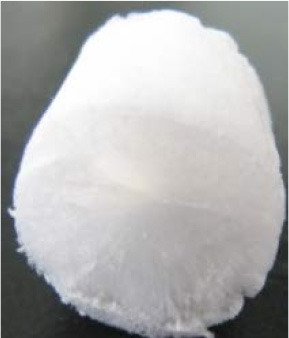

The oxidation of the BC allowed shrinkage of the BC aerogel to be reduced from 41 to 17.8%. It is worth noting that the OD of the BC affected the amount of the aerogel shrinkage. At the ODs of 1.44 and 3.04%, the shrinkage of the aerogel was minimal (17.8%). At the minimum OD of 1.03%, the aerogel shrinkage value was 31.62%, which was lower than that of the control sample of the NBC aerogel. An increase in the OD of the BC to 4.15% led to an increase in the aerogel shrinkage from 17.8 to 21.27%. Thus, the obtained results indicate an optimal influence of BC OD is possible on the aerogel shrinkage during its production through the freeze-drying process.

**Figure 4:**
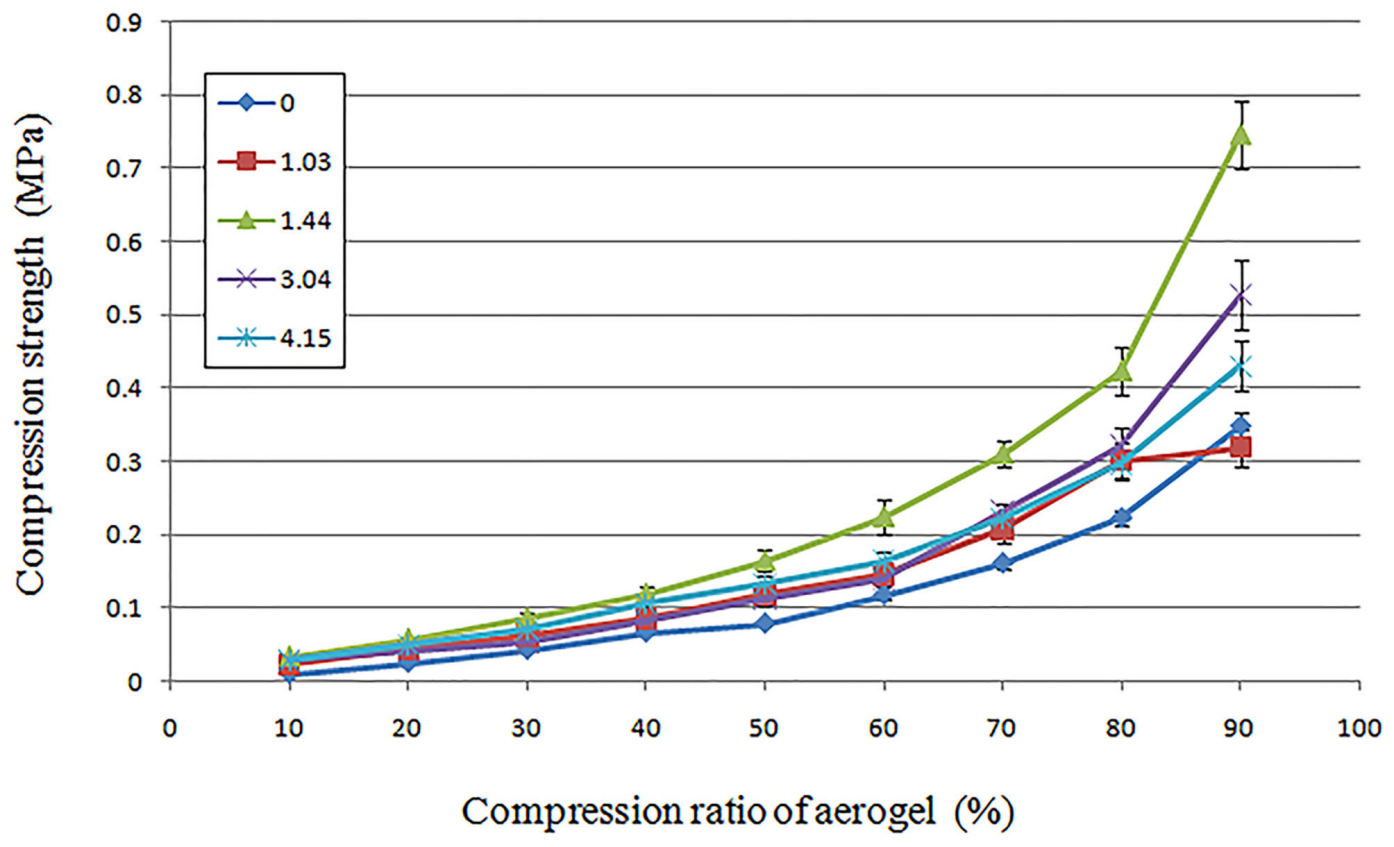
Effect of the ODs of BC on the strength properties of the produced aerogels.

The study of the strength of the OBC aerogels showed that the TEMPO oxidation of the BC led to a change in the strength of the aerogels (Figure 4).

Compared to the control aerogel obtained from NBC, the aerogel obtained from the OBC, which had an OD of 1.44%, exhibited the most pronounced effect. Compressing an aerogel sample from the BC with an OD of 1.44% required twice strength compared to an aerogel sample obtained from the NBC. An increase in the OD to 3.04 and 4.15% led to a decrease in the strength of the OBC aerogels, which was nevertheless higher than the NBC control aerogels.

### Effect of Divalent Metals on the Strength of Aerogels Made of Oxidized BC

**Figure 5:**
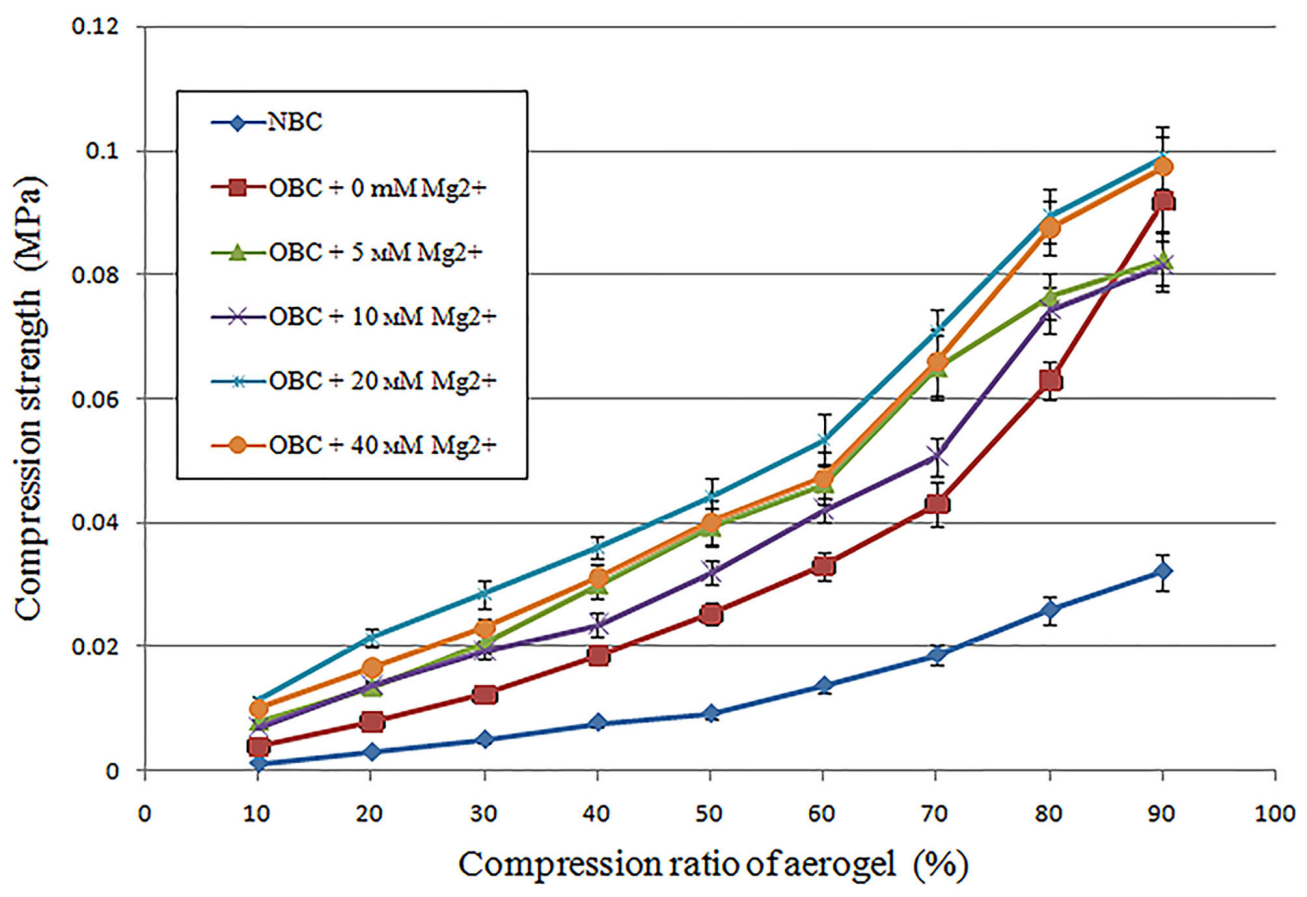
Changes in the strength properties of the aerogels obtained from the TEMPO-oxidized BC (OBC), with an OD of 1.44% depending on the concentration of Mg^2+^, compared with the aerogel obtained from the NBC.

The introduction of the–COOH groups in the BC by TEMPO oxidation enables further increase in the strength properties of the OBC aerogels by forming cross-links between two –COOH groups with help of divalent cations. For this purpose, Mg^2+^of specific concentration was added to the OBC hydrogel with an OD of 1.44%, and the strength of the obtained samples was compared (Figure 5).

The addition of Mg^2+^ resulted in an increase in the compressive force applied to the aerogel sample to achieve a certain degree of the sample compression. The largest increment in the compressive force on an OBC aerogel sample with an OD of 1.44% was achieved when Mg^2+^ was added at concentrations of 20 and 40 mM. To achieve 50% sample compression of the samples with an OD of 1.44% containing 20 or 40 mm Mg^2+^, compressive forces four- and five-fold higher than those applied on the NBC aerogel were required.

**Figure 6:**
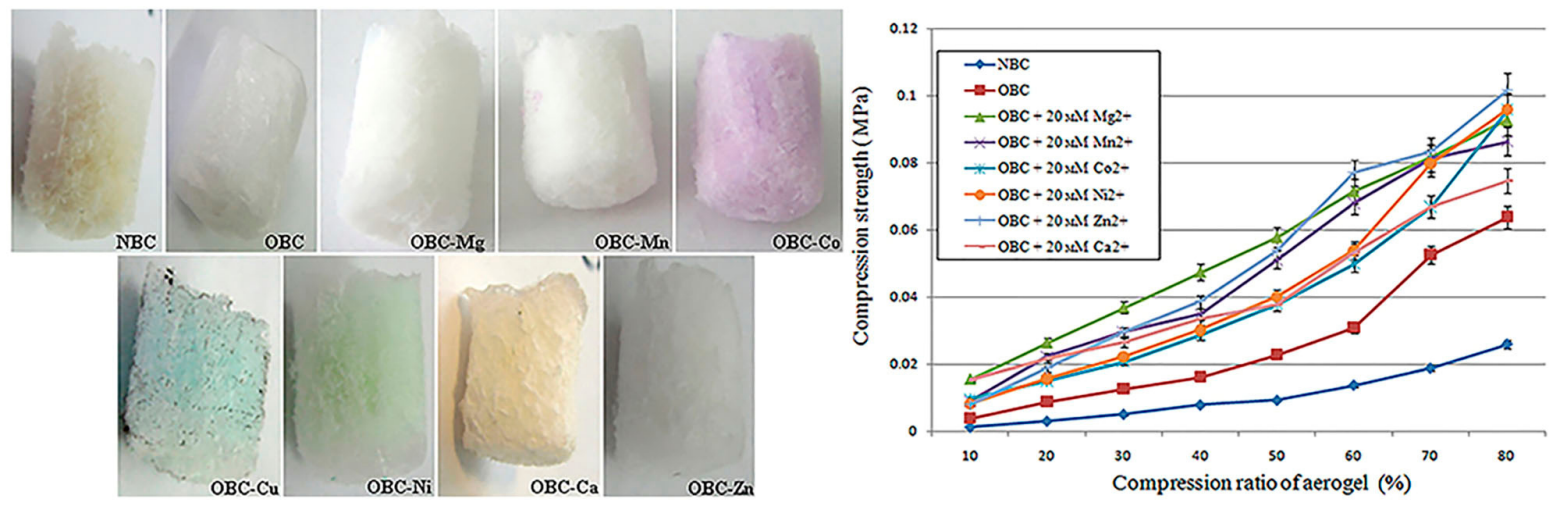
Samples of BC aerogels and changes in their strength. **(A)** BC aerogels produced from NBC and TEMPO-oxidized BC with an OD of 1.44% with presence of 20 mM of corresponding divalent metals: Mg^2+^, Mn^2+^, Co^2+^, Cu^2+^, Ni^2+^, Ca^2+^, and Zn^2+^. **(B)** Changes in the strength of the aerogels prepared from the TEMPO-oxidized BC with an OD of 1.44% depending on the presence of 20 mM of divalent metals.

Owing to the fact that the introduction of Mg^2+^ into the BC aerogel improved the strength properties of the material, the influence of other divalent ions, such as Mn^2+^, Co^2+^, Cu^2+^, Ni^2+^, Ca^2+^, and Zn^2+^, on the strength of the OBC aerogels with an OD of 1.44% was also evaluated. These ions were added to the BC hydrogels in the amount necessary to achieve a final concentration of 20 mM (Figure 6A). The changes in strength properties were determined for the obtained aerogels (Figure 6B).

Before achieving 50% sample compression, the OBC aerogel with Mg^2+^ ions was observed to be the most durable aerogel. At a sample compression of 60–80%, the strength of the aerogels containing Mg^2+^ions was comparable to that of the aerogels containing Zn^2+^or Mn^2+^ ions. Further, Co^2+^, Ni^2+^, and Ca^2+^ had the least influence on the strength of the aerogels. However, all the metals increased the strength of the aerogels prepared from the TEMPO-oxidized BC. The simplicity of this approach is primarily attributed to the use of divalent ions as a cross-linking agent, which does not require further modification of BC, because chemical modification always destroys the weak interactions that play a major role in aerogel stabilization.

Thus, oxidation of the BC solved several problems at once in the process of obtaining an aerogel from the BC. The oxidation of the BC resulted in reduced shrinkage of the OBC aerogel, and increased the strength of the OBC aerogels, which can be further enhanced by the introduction of divalent metal ions.

### Microporous Structure of Aerogels Obtained From NBC or OBC

Utilizing the TEMPO-oxidized BC to produce aerogels resulted in both a decrease in the aerogel shrinkage and an increase in the porosity and strength of the material. Obviously, these changes are correlated to the microporous structure changes of the BC aerogels. The porous structures of the aerogels obtained from the NBC and the TEMPO-oxidized BC with an OD of 1.44% were studied, in the absence or presence of 20 mM Mg^2+^. The microporous structure in the central and outer parts of the aerogels was studied using cylindrical aerogels with a diameter of 14 mm and a height of 10 mm.

**Figure 7:**
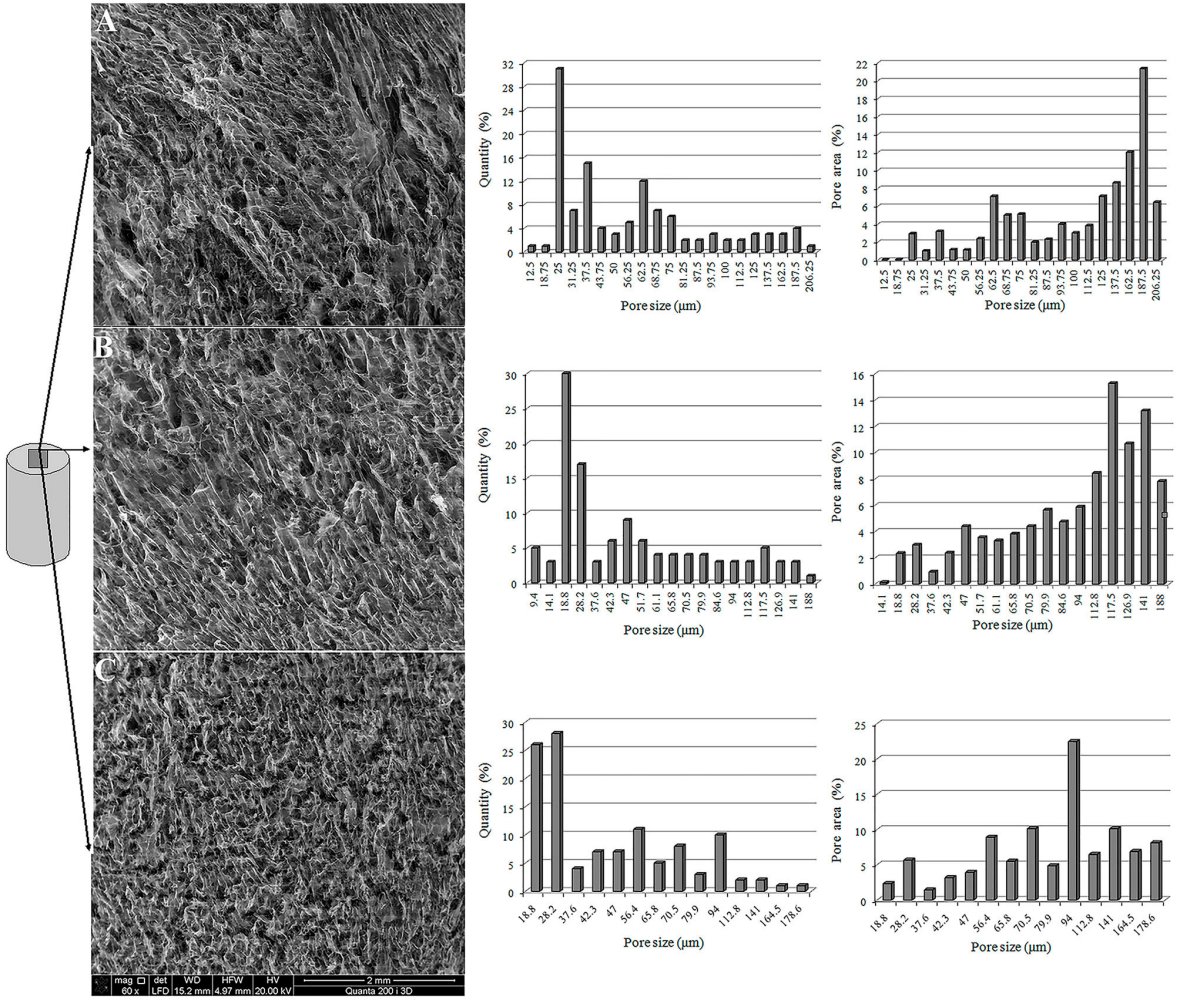
SEM image of the central part of the aerogels prepared from the NBC **(A)**, TEMPO-oxidized BC with an OD of 1.44% **(B)**, and TEMPO-oxidized BC with an OD of 1.44% containing 20 mM Mg^2+^**(C)**.

In the central part of the aerogels obtained from the NBC or OBC, no significant change in the microporous structure was observed; however, the aerogel obtained from TEMPO-oxidized BC has a tendency to increase the number of smaller pores. The oxidation of the BC reduced the maximum pore size in the central part of the aerogel from 206 to 188 μm (Figure 7). This reduction is not significant; however, the total area of the pores, the size of which does not exceed 100 μm in the aerogel from TEMPO-oxidized BC, increased from 40.6 to 44.6%. The addition of 20 mM Mg^2+^ to the aerogels obtained from the TEMPO-oxidized BC further reduced the maximum pore size to 175.6 μm. Simultaneously, the area of the pores, with pore sizes <100 μm, in this aerogel increased to 68.4%. Thus, the central part of the aerogels obtained from the TEMPO-oxidized BC, particularly with the Mg^2+^ ions, was characterized by a more developed porous structure that has a decreased maximum pore size and an increased number of smaller pores.

**Figure 8:**
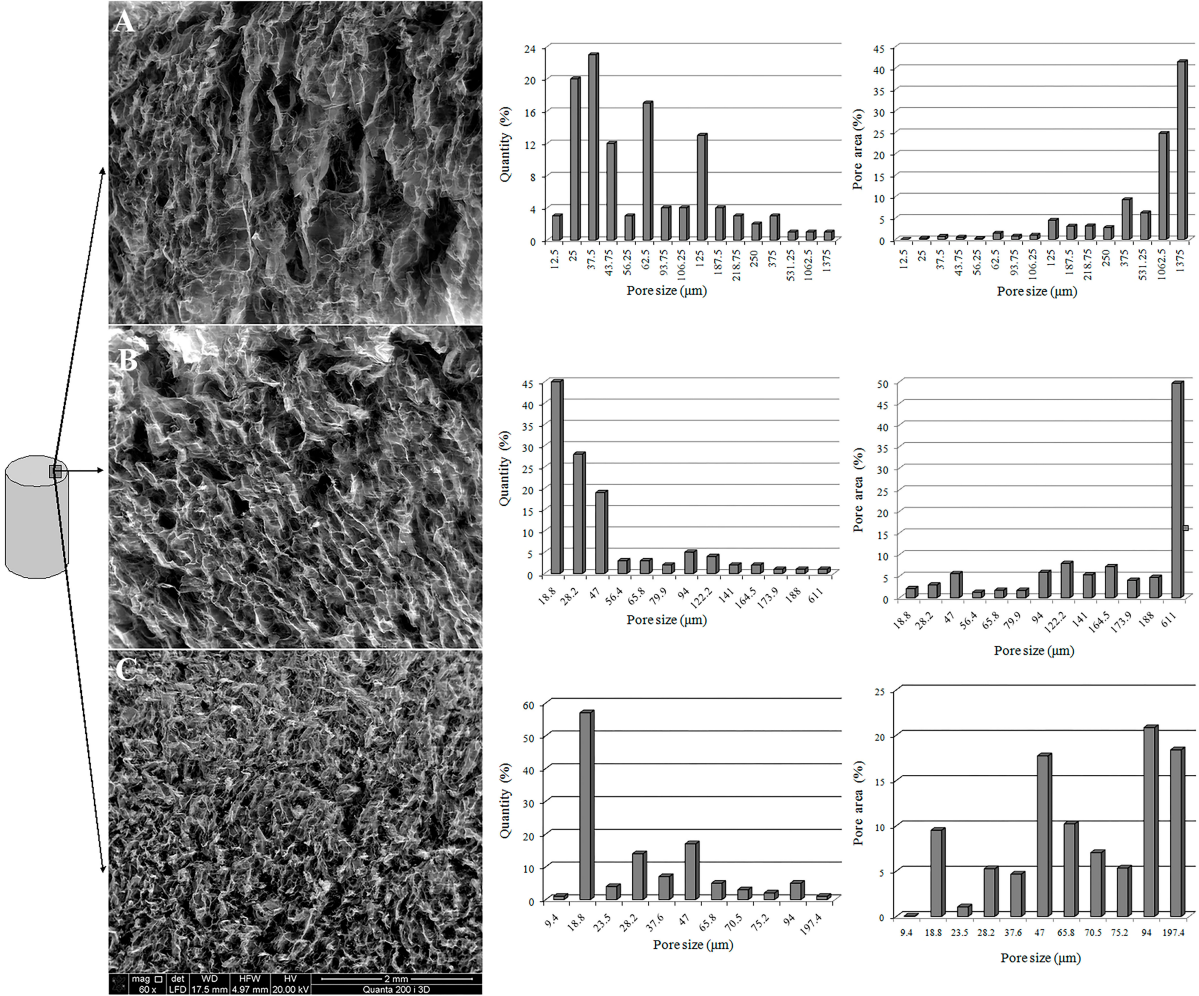
SEM image of the outer part of the aerogels prepared from the NBC **(A)**, TEMPO-oxidized BC with an OD of 1.44% **(B)**, and TEMPO-oxidized BC with an OD of 1.44% containing 20 mM Mg^2+^**(C)**.

In contrast to the central part of aerogels, the porous structures of the outer part of the aerogels prepared from the NBC or TEMPO-oxidized BC varied significantly (Figure 8). The NBC aerogels had a significant number of pores larger than 1 mm, and these large pores account for most of the occupied area and volume. For example, the proportion of the pores with sizes < 100 μm in the NBC aerogel accounts for up to 70.7% of their total number. However, these pores occupy only 3.9% of the total pore area. Utilizing the TEMPO-oxidized BC reduced the maximum average pore size from 1,375 to 611 μm. The amount of pores with pore sizes <100 μm in the aerogel obtained from the TEMPO-oxidized BC with an OD of 1.44 was 90.5% of the total number of pores, and the total area occupied by these pores increased to 21.2% of the total pore area. Despite the decrease in the average pore size of the TEMPO-oxidized BC aerogel, the maximum pore size in the outer part of the aerogel was three times larger than in the central part. The addition of 20 mM Mg^2+^ to the TEMPO-oxidized BC aerogel resulted in a noticeable reduction in the average pore size compared to the pore sizes of the NBC aerogels. The microporous structure of the outer part of the aerogel was like that of its central part. The average maximum pore size was 197.4 μm. In the TEMPO-oxidized BC aerogel with an OD of 1.44% containing Mg^2+^, the proportion of pores with pore sizes < 100 μm was 99.1%, and the total area occupied by these pores increased to 81.6%.

These results suggest that the introduction of negatively charged groups, such as –COOH, into the BC enabled the stabilization of the BC hydrogel because of their mutual repulsion during freeze-drying. The addition of Mg^2+^ during the preparation of the aerogel from the TEMPO-oxidized BC stabilized the fibrils of the BC in the freezing process more effectively through the ionic bonds between the –COOH groups and the Mg^2+^ ions.

### Determination of Thermal Properties of Aerogels Obtained From Native or Oxidized Bacterial Cellulose

**Figure 9:**
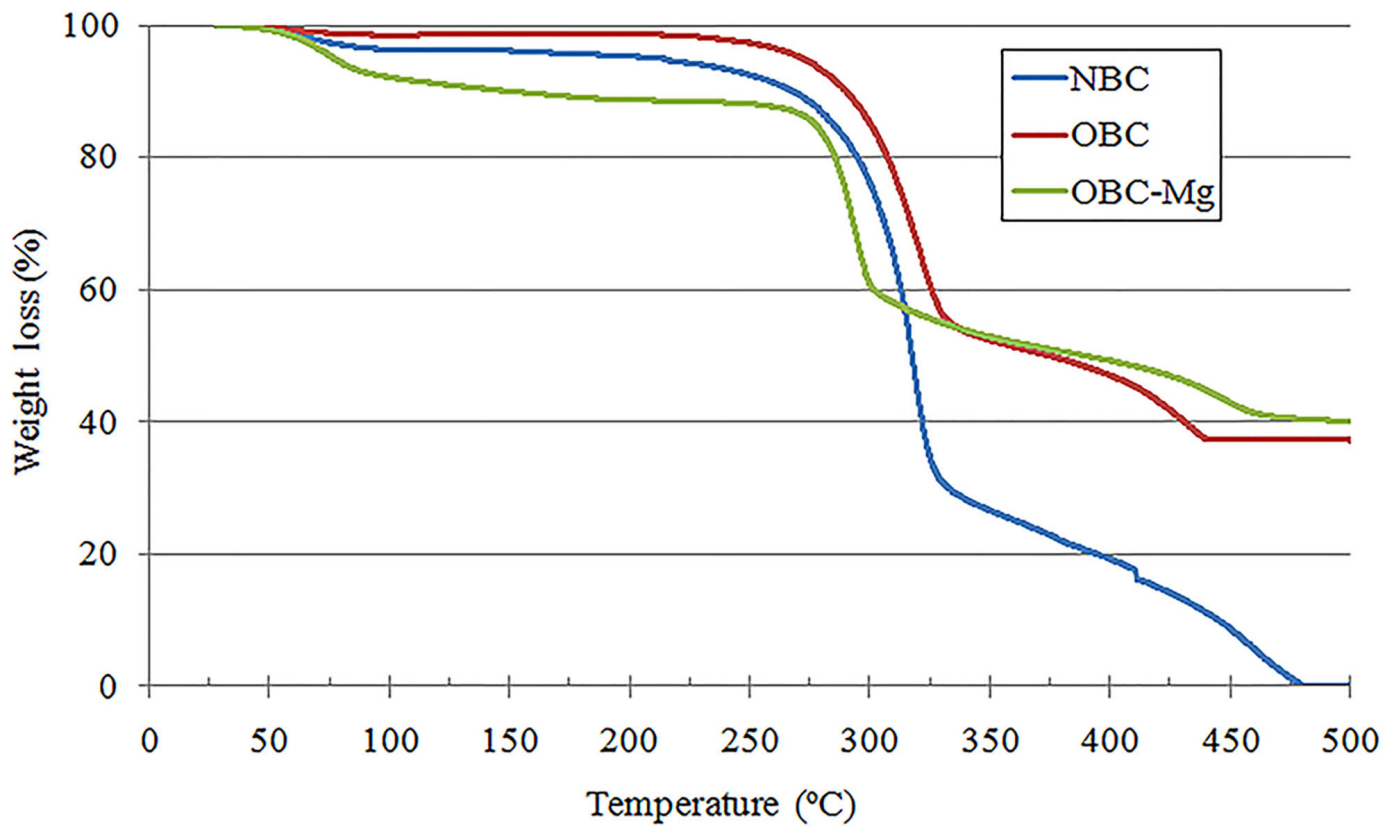
Thermogravimetric analysis of BC aerogels produced from the NBC, TEMPO-oxidized BC with an OD of 1.44% with or without of 20 mM of Mg^2+^.

The temperature stability of BC aerogels is an important characteristic, especially in high-temperature applications of this material. Therefore, we conducted a thermogravimetric study of BC aerogels produced from the NBC and TEMPO-oxidized BC with an OD of 1.44% with or without of 20 mM of Mg^2+^ (Figure 9). Generally, NBC aerogel shows initial decomposition in one step ranging from 275 to 330″C The weight loss of the NBC aerogel started at 57°C, and upon reaching a temperature of 250°C, the NBC aerogel lost 7.5% of its mass. This weight loss is caused by the evaporation of bacterial-cellulose-related water (Huang et al., 2014). Thermogravimetric analysis showed that the thermal decomposition of the NBC aerogel began at 275°C, and by the time the temperature reached 330°C, there was a loss of 70% of the NBC aerogel mass in the oxidizing atmosphere. This result is consistent with thermogravimetric characteristic of the thermal stability of BC film (Revin et al., 2019). The main mass loss in the OBC aerogel or OBC-Mg aerogel also began at 275°C and continued till 330 and 300°C, respectively. The mass loss under these conditions was 43% and 39% for the OBC aerogel and OBC-Mg aerogel, respectively. When the temperature reached 500°C, the mass loss for the OBC and OBC-Mg aerogels was 63 and 60%, respectively, while for the NBC aerogel, the mass loss at this temperature was 99%. The results show that the TEMPO oxidation of BC does not lead to changes in the thermal stability of the material however, the nature of the thermal degradation of the material depended on the oxidation of BC and the presence of Mg^2+^.

Owing to the changes in the microporous structure of the aerogels from the TEMPO-oxidized BC, and owing to the fact that the aerogel obtained from the BC is a highly porous material that can be used as a heat-insulating material, it was interesting to determine how the oxidation of the BC will affect the thermal conductivity of this material. To understand this, we compared the coefficients of thermal conductivity of the aerogels obtained from the NBC, TEMPO-oxidized BC, and TEMPO-oxidized BC containing Mg^2+^ (Table 2). While measuring the coefficient of thermal conductivity, the temperature on the warm side of the sample was 50°C and that on the cold side of the sample was 40°C. Under these conditions, the coefficient of thermal conductivity of air was 0.0283 W/(m•K). The aerogel obtained from the NBC had the highest coefficient of thermal conductivity, most likely because of the presence of the heterogeneous porous structure of the aerogel, which facilitated the transfer of heat. Utilizing the TEMPO-oxidized BC to produce aerogels made it possible to obtain a material with a more uniform porous structure over the entire volume of the sample as compared to the native cellulose aerogel. This led to a decrease in the thermal conductivity coefficient from 0.036 to 0.0225 W/(m•K). The preparation of the aerogel from the TEMPO-oxidized BC with an OD 1.44% and Mg^2+^ led not only to an increase in the strength of the aerogels and a more uniform porous structure, but also to a further decrease in the coefficient of thermal conductivity to 0.0176 W/(m•K).

**Table 2:** Coefficients of thermal conductivity of the aerogels obtained from the NBC and the TEMPO-oxidized BC.

**Aerogels prepared from:**	**Coefficient of thermal conductivity, W/(m·K)**
NBC	0.036
TEMPO-oxidized BC with oxidation degree 1.44%	0.0225
TEMPO-oxidized BC with oxidation degree 1.44%, 20 MM Mg^2+^	0.0176

### Preparation of Aerogels With Antibacterial Properties Based on Native and Oxidized BC

The aerogels obtained in this work can be used as functional biomaterials for a wide range of applications, particularly in biomedicine. This work shows that the strength of an aerogel with an OD of 1.44% doubled compared to that of the aerogels obtained from the NBC. Therefore, we used the TEMPO-oxidized BC with an OD of 1.44% and NBC as a control to obtain antibacterial biocomposites.

The BC has many characteristics of an ideal wound dressing. However, in the native state, it does not show any antimicrobial effects. To produce aerogels with antibacterial properties, we used the antibiotic SF, which is gaining interest in the treatment of skin infections (Siala et al., 2018). Fusidic acid (FA) is used to treat a variety of infections caused by Gram-positive bacteria and is particularly effective against infections caused by staphylococci, including strains resistant to penicillin and other antimicrobials as an alternative for the treatment of infections caused by methicillin resistant *Staphylococcus aureus* (MRSA). In addition, it is a hypoallergenic drug with low toxicity, low drug resistance, and no cross-resistance with other clinically used antibiotics (Curbete and Salgado, 2016).

**Figure 10:**
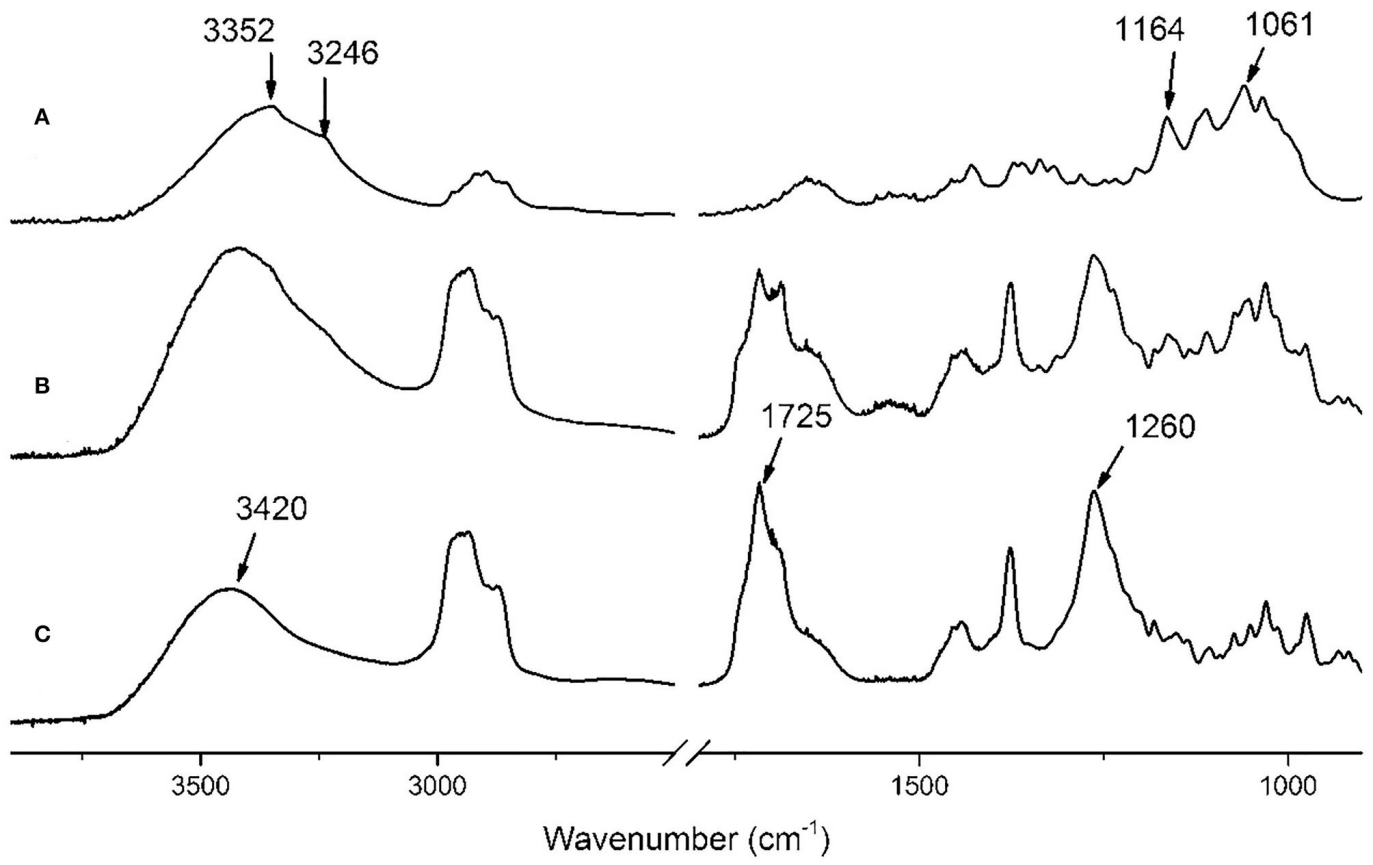
FTIR spectra of the aerogels: NBC **(A)**, NBC/SF **(B)**, and SF **(C)**.

In the present study, novel biocomposites in the form of aerogels based on the NBC and SF (NBC/SF) and the TEMPO-oxidized BC and SF (OBC/SF) with antibacterial activities were obtained for the first time by the incorporation of SF with the NBC and the OBC hydrogel. Figure 10 shows the FTIR spectra of the NBC, SF and NBC/SF composite. The typical FTIR spectrum of the NBC for this polymer is shown, where the absorption in the region of 3,200–3,500 cm^−1^ is due to the valence vibration of the hydrogen-bonded OH groups (Shao et al., 2016). In the C–O stretching vibration region, the peaks at 1,164, and 1,061 cm^−1^ correspond to the C–O–C asymmetric stretching and the C–C, C–OH, C–H ring and side group vibrations, respectively. The infrared spectrum of pure SF exhibited three characteristic bands at 3,420, 1,725, 1,260 cm^−1^. The absorption at the 3,420 and 1,725 cm^−1^ were caused by the valence vibration of the hydrogen-bonded OH groups and carboxyl groups of the SF. In the case of the NBC/SF composite, the FTIR spectrum showed typical bands for the NBC and SF at 1,725 and 1,260 cm^−1^. The presence of these peaks at the FTIR spectrum of the NBC/SF indicates the presence of the SF in the obtained composite.

**Figure 11:**
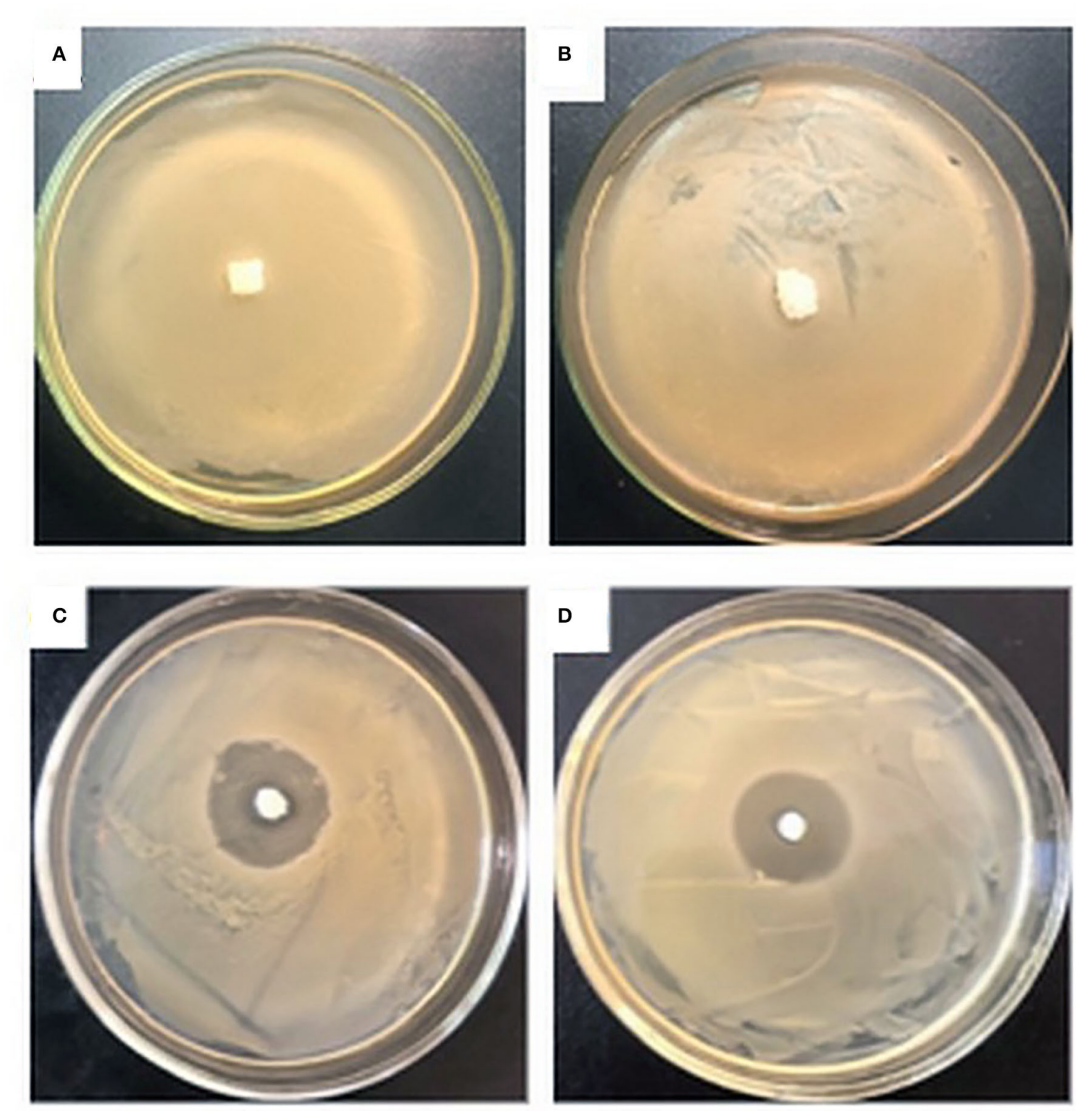
Antimicrobial activities of aerogels against *S. aureus*: NBC **(A)**, OBC **(B)**, biocomposite NBC/SF_100_ **(C)**, biocomposite OBC/SF_100_ **(D)**.

The antibacterial activities of the prepared aerogels were studied by the disc diffusion method. The antibacterial capacity was determined by measuring the diameter of the clear zone of the inhibition around the samples after 18 h incubation, as shown in Figure 11.

As expected, no inhibition zones were observed for the NBC as control, or the OBC, demonstrating that the NBC and OBC do not have any antibacterial abilities. The biocomposites NBC/SF_100_ and OBC/SF_100_ exhibited high antibacterial activities, and the diameters of their zones of inhibition for *S. aureus* were 28 ± 1 and 27 ± 1 mm, respectively. This study clearly illustrates that the prepared aerogels show excellent antibacterial activities. There was no significant difference to be found between the NBC/SF and OBC/SF aerogels. However, as shown in our study, the aerogels obtained from the OBC have greater strength and the functional biomaterials obtained from it will have a wide range of applications, particularly for biomedicine.

**Figure 12:**
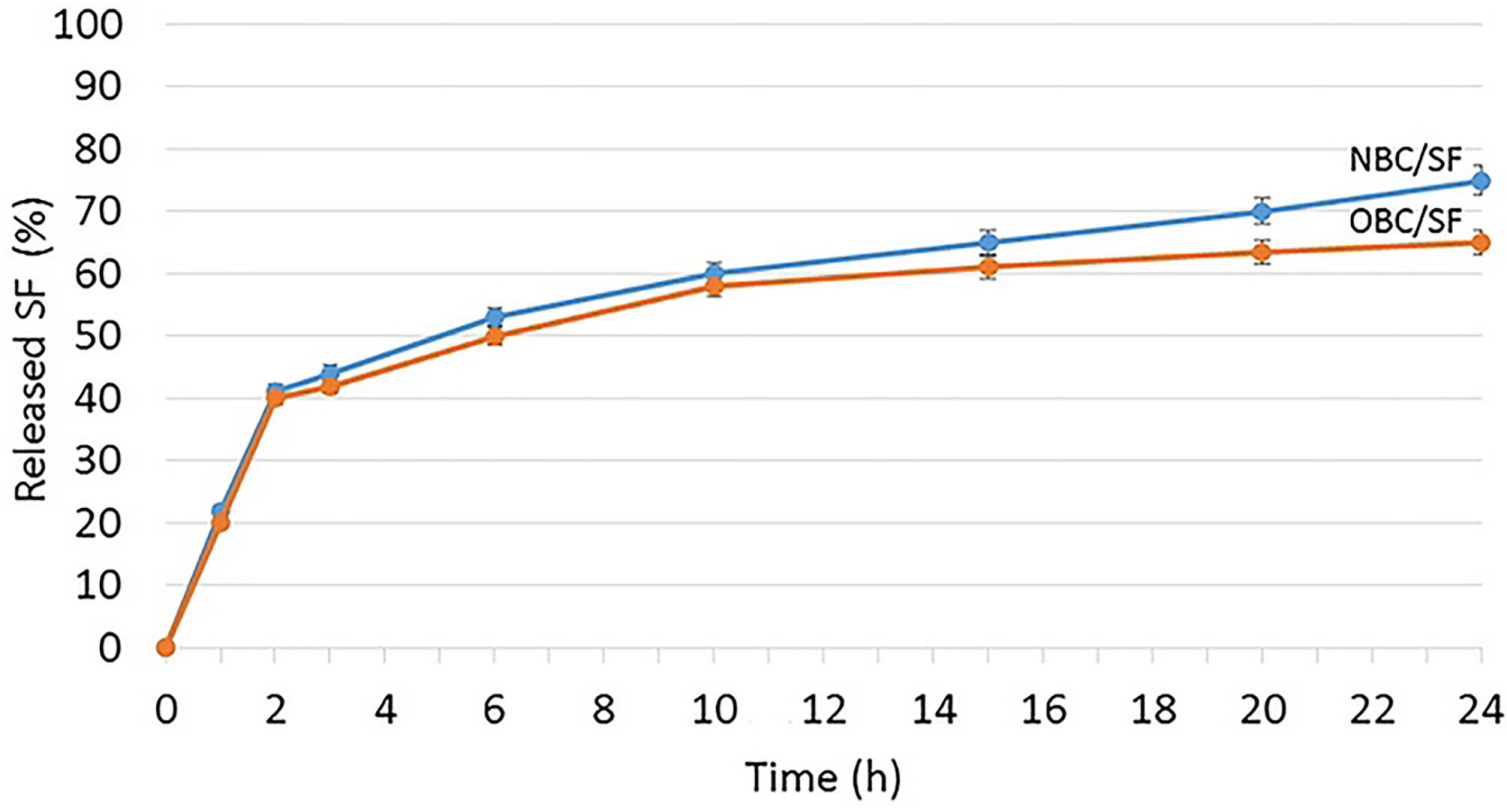
Cumulative release profiles of SF from NBC/SF and OBC/SF biocomposites.

For an antimicrobial material, the release ratio of the antimicrobial agent is important for its practical applications. An antimicrobial agent exhibiting optimally stable and prolonged release is favored. In this study, SF was adsorbed on the nanofibres of NBC and OBC. To study the release behavior of aerogels loaded with SF, the samples were placed in a phosphate buffer solution at pH 7.4 for observation. As shown in Figure 12, the release amount changed with the increase in the sustained release time. It was found that the amount of drug released from NBC/SF and OBC/SF reached 75% and 65% in 24 h, respectively. SF loaded into NBC and OBC can obviously slow the release activity, which suggests that NBC and OBC could be used for the controlled release of drugs that reduce infection and inflammation. The drug release results showed that cellulose aerogels have controlled SF release performance.

**Figure 13:**
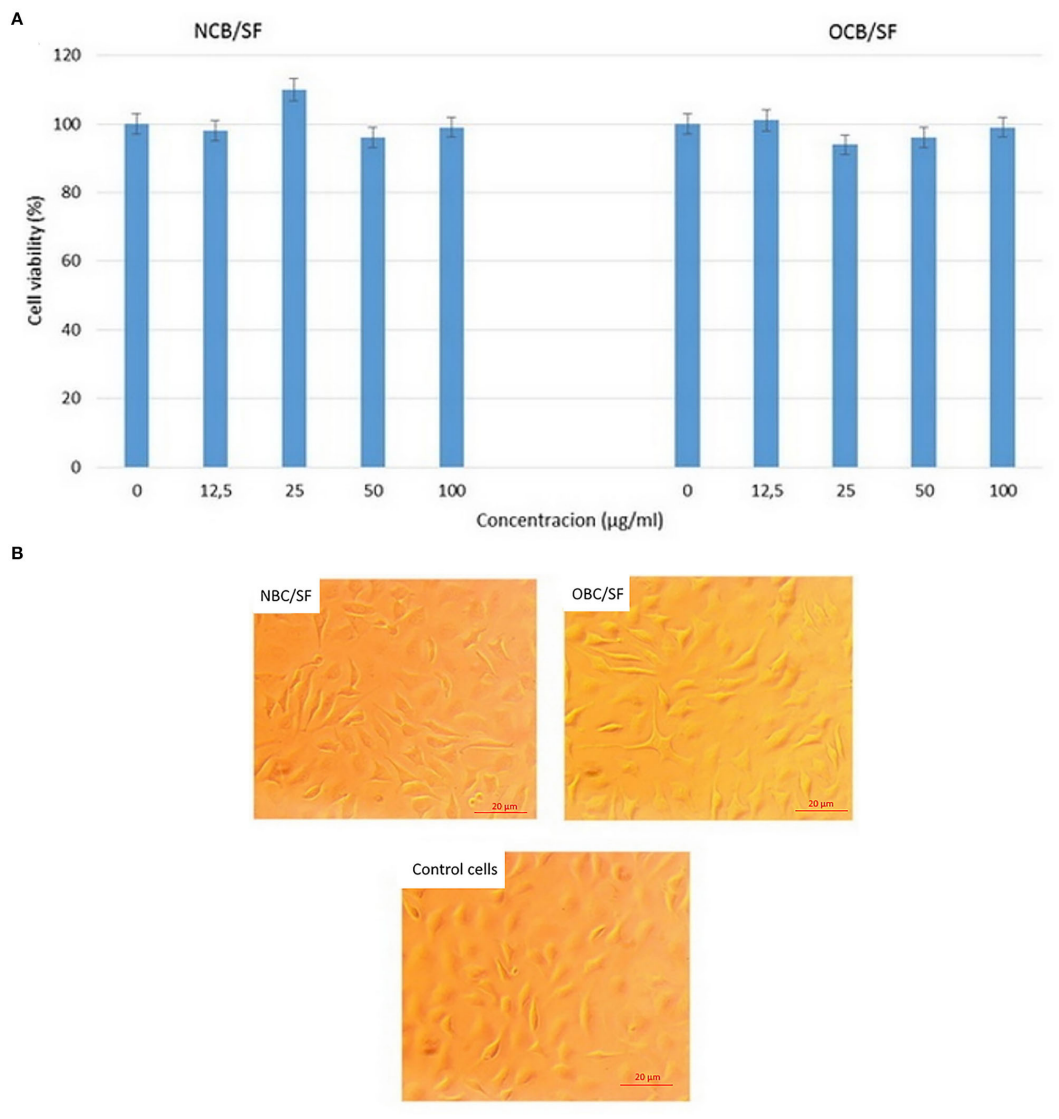
Cell viabilities **(A)** and cell morphologies **(B)** of the mouse fibroblast cell culture L929 treated with NBC/SF and OBC/SF extracts.

Cytotoxicity studies were performed to investigate the effect of SF in the NBC and OBC matrix on proliferation of mouse fibroblast cell culture L929. The cell viability of L929 cells was evaluated by MTT assay. The cell cytotoxicity imparted by NBC/SF_100_ and OBC/SF_100_ aerogels extracts was studied. The MTT results are shown in Figure 13A. All the materials showed negligible toxicity. No reduced cell viability following their incubation with NBC/SF and OBC/SF extracts was shown. The results showed that SF does not inhibit the proliferation of L929 cells. To determine whether the morphology of L929 cells was affected by NBC/SF and OBC/SF biocomposites, the cells were observed by an inverted optical microscope (Figure 13B). The morphologies of L929 cells treated with NBC/SF and OBC/SF extracts were similar to the blank one. Thus, the prepared biocomposites displayed no effect on L929 cells morphology. These results showed that NBC/SF and OBC/SF biocomposites are promising candidates for wound dressing and tissue engineering applications.

## Discussion

The utilization of the freeze-drying method for preparing aerogels from BC indicates the presence of a step for pre-freezing the hydrogel of BC. This step will ultimately determine the microporous structure of the aerogel, which, in turn, will determine the aerogel properties such as thermal conductivity, strength, and porosity (Revin et al., 2019). Obviously, the freezing process will not only be determined by the freezing mode but also will depend on parameters such as volume of the frozen sample. It is known that the production of aerogels from BC by freeze-drying is accompanied with their shrinkage, which is expressed as a decrease in their volume, a change in porosity (as observed in our studies), and the existence of heterogeneity of the microporous structure in different sections of the aerogel. According to our data, the shrinkage volume can be significant and reach 40% or more (Table 1). Moreover, the central part of the aerogel obtained from BC was denser than the aerogel sections located on the outer part of the sample. The presence of such heterogeneity in the structure will undoubtedly become more pronounced with an increase in the volume of the aerogels, and will make it difficult to design functional materials based on them.

It is possible that the shrinkage of the aerogel may be caused by the freezing-out of the material during its freezing. Accordingly, prevention of such an undesirable phenomenon by changing the properties of BC is possible. It is known that the chemical modification of cellulose is widely conducted to change the properties of this polymer as well as to overcome some difficulties, for example barrier properties, in preparing functional materials (Aulin et al., 2010; Habibi et al., 2010; Rodionova et al., 2010; Kang et al., 2015).

Because it is assumed that the formation and stabilization of the structures of the BC hydrogel and the aerogel obtained from it are dependent on the formation of hydrogen bonds, the introduction of additional functional groups into this system could lead to changes that would reduce shrinkage and increase the homogeneity of the microporous structure of aerogels. The role of such additional functional groups is well-aligned with the carboxyl group which can easily be introduced into BC during its TEMPO oxidation. Our results showed that the introduction of carboxyl groups into BC resulted in a decrease in the shrinkage of aerogels from 41.02 to 17.8% and an increase in the strength of this material (Table 1, Figure 4). Moreover, the nature of these changes was directly dependent on the number of carboxyl groups introduced. It is assumed that a decrease in the shrinkage observed with the introduction of carboxyl groups into BC could be due to mutual repulsion of negatively charged carboxyl groups, which, in turn, could lead to the stabilization of the hydrogel of BC during its freezing at a given OD. An excessive increase in the OD, resulting from the introduction of a large number of carboxyl groups, could lead to the destruction of the system of stabilizing hydrogen bonds, which negatively affects both the shrinkage and strength of the aerogel. The results obtained on the effect of chemical modification on the such parameter as shrinkage are in good agreement with the data on the effect of chemical modification on the properties of functional materials from BC obtained in the studies of other authors (Aulin et al., 2010; Habibi et al., 2010; Rodionova et al., 2010; Kang et al., 2015).

Thus, the oxidation of BC solved several problems associated with the process of obtaining aerogel from BC. First, the oxidation of BC resulted in reduced shrinkage of the aerogels from OBC. Secondly, the oxidation of BC led to an increase in the strength of the aerogels from OBC. This high strength of BC aerogels is extremely advantageous for applications such as a thermal insulator or in the fabrication of a composite material for medical purposes. According to our data, the aerogels from BC with a lower density had a lower coefficient of thermal conductivity (Revin et al., 2019). Moreover, although the strength of the BC aerogels significantly decreased with a decrease in their density, for this field of application, it is necessary to maintain a high strength of the material at a low density. This problem can be solved by the introduction of additional cross-links. In this work, divalent metal ions were used to create cross-links between two carboxyl groups located close to each other. Thus, the inclusion of 20 mM Mg^2+^ in the aerogel resulted in a four- to five-fold increase in the strength of aerogels prepared from TEMPO-oxidized BC compared to that of aerogels prepared from NBC (Figure 5). The use of divalent ions as a cross-linking agent was primarily due to the simplicity of this approach, which does not require further modification of BC, because chemical modification always destroys the weak interactions that play a major role in aerogel stabilization. However, the development of the material is also based on its applications. The use of these aerogels as a functional element of a structural material, such as a heat-insulating material (because it has a low coefficient of thermal conductivity, as shown in this study), would be possible. Moreover, the aerogels can be used as a sound-insulating material, because it has sufficiently high sound absorption coefficients in the region of 1,600–5,000 Hz (Revin et al., 2019). However, in either of these applications, the use of divalent metals related to heavy metals will be impossible for environmental reasons. In contrast, when preparing medical materials, the use of divalent metals including heavy metals may be advisable because some of them have pronounced antimicrobial properties (Robinson et al., 2010; Argueta-Figueroa et al., 2014).

The inclusion of Mg^2+^ in the composition of the aerogel from TEMPO-oxidized BC resulted in a more uniform microporous structure of the aerogel with maximal pore sizes in the central and outer parts of the aerogel 178 and 197 μm, respectively, unlike the structure of aerogel prepared from NBC (for which the maximum pore size varied from 206 μm in the central part of the aerogel to 1,375 μm in the outer part of the sample). Apparently, changing of the aerogel microporous structure caused a decrease in the thermal conductivity coefficient of the aerogel from TEMPO-oxidized BC containing Mg^2+^ from 0.036 to 0.0176 W/(m•K) (Table 2) compared to that of the aerogel from NBC.

Thermogravimetric analysis has showed that the oxidation of BC or the inclusion of Mg^2+^ in the aerogel from OBC did not lead to a change in the temperature at the beginning of thermal degradation of the material, but the pattern of change of thermal degradation of the aerogels depended on the oxidation of BC and the presence of Mg^2+^. This may be due to the fact that the number of carboxyl groups included in BC was not large enough, since the calculation of the percentage of carboxyl groups reflects only the number of primary CH_2_OH groups subjected to oxidation. It is necessary to note that the thermal stability of the obtained aerogel will only be important in certain potential applications of this material. There is a large opportunity for creating materials for medicine, including those based on BC, where in thermally stable materials are not required.

The resulting aerogels can be used as functional biomaterials in a wide range of applications, particularly in biomedicine. Recently, aerogels were incorporated in the field of biomaterials (Nita et al., 2020) because of their properties such as high porosity, high internal surfaces, and controlled pore diameter and their 3D interconnected structure. Bio-based aerogels also exhibit high cytocompatibility, biocompatibility, and biodegradability and can be used successfully in biomedical applications such as tissue engineering and drug delivery and as antibacterial materials (El-Naggar et al., 2020; Nita et al., 2020). In this study, the TEMPO-oxidized BC with an OD of 1.44% and NBC were used to produce antibacterial biocomposites.

The BC has the characteristics of an ideal wound dressing. BC-based dressings have nanofibrous structures with a high capability of absorbing and retaining water and wound exudate and can control the environment for wound healing (Li et al., 2018). Therefore, BC must be considered as a physical barrier between the wound and the surrounding environment, thereby preventing microbial infections. There have been several successful attempts to impart antimicrobial properties to BC. BC-silver nanocomposites exhibited excellent antibacterial activity (Wu et al., 2018; Wang et al., 2020). The synthesis of BC/chitosan composites with high mechanical reliability and antibacterial activity has also been reported (Zang et al., 2016). Aerogels with antimicrobial properties based on chitosan (Zheng et al., 2020) and cellulose with amoxicillin (Ye et al., 2018) have also been reported.

Among the technologies for constructing antibacterial materials, adding antibiotics in the BC is a facile method to obtain an antibacterial film that can be used as a wound dressing material (Kaplan et al., 2014). Previously, we obtained biocomposites with high antibacterial activity based on BC and FA in the form of films (Liyaskina et al., 2017). FA, an antibiotic produced from the *Fusidium coccineum* fungus, belongs to the class of steroids, but it has no corticosteroid effects (Liu et al., 2020). FA is characterized as an α, β-unsaturated carboxylic acid; its molecular formula is C_31_H_48_O_6_, containing one acetoxyl and two hydroxyl groups. The most active derivative is its sodium salt (sodium fusidate), which was first used clinically for the treatment of staphylococcal infections. FA has been widely used in the systemic and topical treatments of staphylococcal infections, including those caused by strains resistant to penicillin and other antimicrobials, thereby making it an alternative for the treatment of diseases caused by the MRSA strains (Fernandes, 2016). In recent years, the inhibitory effect of FA on the biofilm formation of *S. aureus* (Siala et al., 2018) was reported. FA can effectively reduce the virulence of *S. aureus* by inhibiting the expression of α-toxin (Liu et al., 2020). Oral FS is currently being re-developed in the US for skin and orthopedic infections, in which biofilms play a major role (Siala et al., 2018).

Drugs can be incorporated into aerogels by two methods (Nita et al., 2020). In the first method, which is considered the simpler method, the drug is added *in situ* during the gelling process. The second method consists of the *ex situ* addition of the drug by absorption or precipitation in a dry aerogel. By this method, the drug in a liquid or gaseous phase is incorporated into the aerogel matrix. However, this latter method has some restrictions; specifically, the slow diffusion capacity of the drug through the pores of the matrix.

In this work, novel biocomposites with antibacterial properties based on native and oxidized BC and SF in the form of aerogels, by including SF in the hydrogels of native and oxidized BC, were obtained for the first time. These biocomposites exhibit high antibiotic activity against *S. aureus* and can be used in medicine as a wound dressing. Aerogels prepared from OBC have high strength and, therefore, are advantageous for use as functional biomaterials for a wide range of applications, including in biomedicine.

## Conclusion

In this study, we developed appropriate conditions for obtaining aerogels with controlled density, strength, and thermal characteristics from BC such that they can be used as functional biomaterials for a wide range of applications, particularly in biomedicine. It was found that the aerogels prepared from OBC are more durable and have a lower aerogel shrinkage compared to the aerogels obtained from NBC. Furthermore, the TEMPO oxidation of the BC along with the addition of Mg^2+^ can produce aerogels with significantly increased strength and a more uniform microporous structure. Furthermore, in this study for the first time, novel biocomposites were formed from aerogels based on either the NBC or the OBC and SF, which had a high antibacterial activity against *S. aureus*. These results can be helpful in the further development of antibacterial wound dressing materials as well as other new antibacterial products for biomedical applications.

## Data Availability Statement

The raw data supporting the conclusions of this article will be made available by the authors, without undue reservation.

## Author Contributions

VR, NP, and EL contributed toward conception and design of the study. ET, NN, and NP performed the experimental work. NP and EL wrote the first draft of the manuscript and contributed toward data analysis and manuscript revision. All the authors have read and approved the submitted version.

## Conflict of Interest

The authors declare that the research was conducted in the absence of any commercial or financial relationships that could be construed as a potential conflict of interest.
